# Spectrum of Genetic Diseases in Tunisia: Current Situation and Main Milestones Achieved

**DOI:** 10.3390/genes12111820

**Published:** 2021-11-19

**Authors:** Nessrine Mezzi, Olfa Messaoud, Rahma Mkaouar, Nadia Zitouna, Safa Romdhane, Ghaith Abdessalem, Cherine Charfeddine, Faouzi Maazoul, Ines Ouerteni, Yosr Hamdi, Anissa Zaouak, Ridha Mrad, Sonia Abdelhak, Lilia Romdhane

**Affiliations:** 1 Laboratory of Biomedical Genomics and Oncogenetics, Institut Pasteur de Tunis, Tunis 1002, Tunisia; nesrine.mezzi@pasteur.utm.tn (N.M.); olfa.messaoud@pasteur.tn (O.M.); rahma.mkaouar@fst.utm.tn (R.M.); zitounanadia@gmail.com (N.Z.); safa.romdhane@pasteur.utm.tn (S.R.); ghaith.abdessalem@pasteur.utm.tn (G.A.); cherine.charfeddine@gmail.com (C.C.); yosr.hamdi@pasteur.utm.tn (Y.H.); sonia.abdelhak@pasteur.utm.tn (S.A.); 2 Department of Biology, Faculty of Sciences of Bizerte, Université Tunis Carthage, Jarzouna 7021, Tunisia; 3 High Institute of Biotechnology of Sidi Thabet, Biotechpole of Sidi Thabet, University of Manouba, Ariana 2080, Tunisia; 4 Department of Congenital and Hereditary Diseases, Charles Nicolle Hospital, Tunis 1002, Tunisia; f.maazoul@yahoo.fr (F.M.); ines_ouertani@yahoo.fr (I.O.); ridhamr@rns.tn (R.M.); 5 Laboratory of Human and Experimental Pathology, Institut Pasteur de Tunis, Tunis 1002, Tunisia; 6 Department of Dermatology, Research Unit Genodermatosis and Cancer LR12SP03, Habib Thameur Hospital, Faculty of Medicine of Tunis, University of Tunis El Manar, Tunis 1002, Tunisia; anissa_zaouak@yahoo.fr

**Keywords:** genetic diseases, Tunisian population, public health, consanguinity, database

## Abstract

Genetic diseases in Tunisia are a real public health problem given their chronicity and the lack of knowledge concerning their prevalence and etiology, and the high rates of consanguinity. Hence, we performed systematic reviews of the literature in order to provide a more recent spectrum of these disorders and to expose the challenges that still exist to tackle these kinds of diseases. A manual textual data mining was conducted using MeSH and PubMed databases. Collected data were classified according to the CIM-10 classification and the transmission mode. The spectrum of these diseases is estimated to be 589 entities. This suggests remarkable progress through the development of biomedical health research activities and building capacities. Sixty percent of the reported disorders are autosomal recessive, which could be explained by the high prevalence of endogamous mating. Congenital malformations (29.54%) are the major disease group, followed by metabolic diseases (22%). Sixty percent of the genetic diseases have a known molecular etiology. We also reported additional cases of comorbidity that seem to be a common phenomenon in our population. We also noticed that epidemiological data are scarce. Newborn and carrier screening was only limited to pilot projects for a few genetic diseases. Collected data are being integrated into a database under construction that will be a valuable decision-making tool. This study provides the current situation of genetic diseases in Tunisia and highlights their particularities. Early detection of the disease is important to initiate critical intervention and to reduce morbidity and mortality.

## 1. Introduction

Rare diseases (RDs) are defined differently from one jurisdiction or organization to another [1]. In the USA, a rare disease (RD) is a disease affecting fewer than 200,000 people in the population (https://rarediseases.info.nih.gov, accessed on 24 September 2021). In Europe, a prevalence of less than 1/2000 people was specified to define a RD [2]. In Japan, any disorder affecting 1 in 2500 people is a RD [3]. In an attempt to define a consensus about an international definition of RD, Ritcher and his colleagues proposed a global average of 40 cases/100,000 people with an average of 1/2500 people [3]. In the Arab world, including Tunisia, there is no standard definition of RD. RDs imply a great global burden and a significant challenge for health systems resulting from unmatched patients’ needs and the absence of equal access to diagnosis and treatment [4]. Nearly 6000 RDs have been identified around the world (https://www.rarediseaseday.org/, accessed on 24 September 2021). Only 5% of them have an FDA-approved treatment (https://globalgenes.org/rare-diseases-facts-statistics/, accessed on 24 September 2021).

Approximately 72% of RDs are attributed to genetic factors (https://www.rarediseaseday.org/, accessed on 24 September 2021). Genetic diseases are generally early-onset and life-threatening conditions [5]. About 30% of affected individuals die before five years [5]. Genetic diseases particularly concern the Arab world, where at least 1890 disorders have been identified (https://www.cags.org.ae/en, accessed on 24 September 2021). Congenital and genetic diseases are a major cause of infant mortality and handicap among Arab countries [6]. The spread of genetic diseases in the Arab world, especially rare conditions, was largely explained by socio-cultural factors [7,8]. Indeed, the highest rates of consanguinity with an extreme prevalence of first-cousin unions were described among Arab families, also known by their large size [9]. In addition, high paternal and maternal age have also been documented among Arabs [6].

Tunisia, a middle-income country, is situated in a strategic position at the crossroad of Europe, the Middle East and Sub-Saharan Africa. The current population is estimated to be 11,860,516 in November 2020, with a population growth rate assessed to 1.11% (https://tradingeconomics.com/tunisia; https://www.worldometers.info/world-population, accessed on 24 September 2021). About 98% of Tunisians are Muslims living with other minorities, such as Christians and Jews [10]. The country has been inhabited since the Paleolithic period [11]. Berbers, also called Amazigh, are the indigenous people of Tunisia [12,13]. This has been well confirmed by the investigation of the HLA class I and class II gene profiles among Tunisians, which shows the Berber origin of the present-day Tunisian population [14]. Throughout its history, the Tunisian population has witnessed successive invasions and several migratory flows during prehistoric and historic periods [13], thus shaping its genetic landscape [10]. 

Tunisia is characterized by the improvement of health system indicators [15]. In the MENA region, Tunisia has the highest life expectancy with remarkable improvements in maternal and infant mortality [16]. This could be explained by the implementation of social programs aiming to reduce the burden of communicable diseases (CD) [15]. Consequently, epidemiological data show that non-communicable diseases (NCD), such as genetic disorders, exceed CD [17]. To a certain extent, this can be explained by changes in national economic activities, family structure, and lifestyle that could foster the rising burden of NCD [18]. Similarly to other Arab countries, familial and geographic endogamy is a deeply conserved rule in Tunisia with remarkable historical and social roots with rates ranging up to 38.0% despite the remarkable socio-demographic changes [7,19,20,21]. This socio-cultural feature has been found to be strongly associated with the expression of rare genetic diseases [19,22]. In a previous study, we reported on the preliminary spectrum of genetic diseases in Tunisia. Such conditions are considered as real problems of public health in Tunisia as their spectrum encompasses 346 genetic disorders [23]. A large proportion is represented by autosomal recessive diseases (62.9%), followed by autosomal dominant diseases highlighting the impact of inbreeding in their emergence [23]. In the present work, we performed a systematic review of the literature relative to genetic diseases in Tunisia in order to (i) “provide a more recent spectrum” of these disorders in the Tunisian population, (ii) identify the type of potential health interventions that could be proposed for these conditions in Tunisia, and (iii) to shed light on challenges that still exist.

## 2. Materials and Methods

### 2.1. Systematic Review of Literature Related to Genetic Diseases Spectrum in Tunisia

A comprehensive review of published data was conducted mainly using the MeSH and PubMed databases. The list of genetic diseases was obtained after querying the MeSH database using the keywords “genetic diseases”. The query results were further combined to additional keywords “tunis*” using the PubMed search tool. We focused only on papers published from 2010 to August 2021. The final query on PubMed was the following: ((“Genetic Diseases, Inborn”[Mesh]) AND (Tunis*)) AND ((“2010”[Date—Publication]: “2021”[Date—Publication])).

The retained papers (*n* = 970) were included in the meta-analysis and were curated manually. They were completely screened for disease name, OMIM ID, inheritance mode, associated gene(s), germinal mutation(s) (according to HGVS nomenclature), and epidemiological parameters (prevalence, incidence, and patient counts). We also searched for the same information from the grey literature (including technical or research reports, doctoral thesis, conference proceedings, etc.). From the selected documents reporting on genetic diseases in the Tunisian population, a recursive search for additional documents was performed using the authors’ names of referring physicians and researchers in Tunisia.

**Figure 1:**
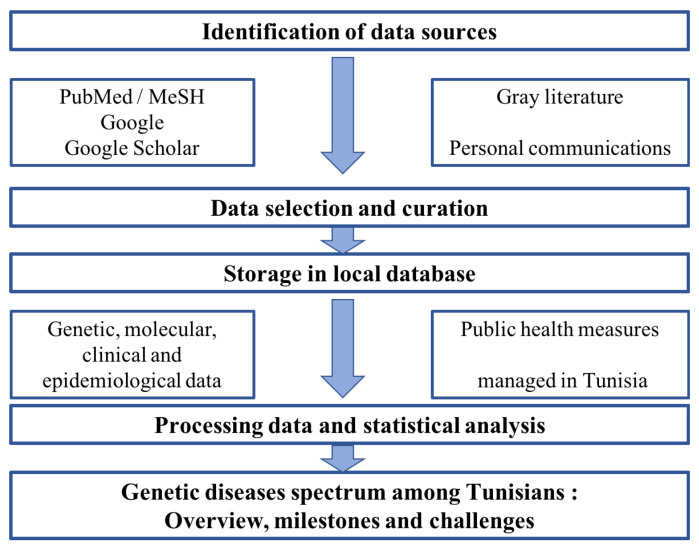
Screening process workflow.

We used the tenth version of the World Health Organization (WHO) International Classification of Disease (WHO ICD-10) in order to provide a clustering of the genetic diseases in different pathological groups. Our previous data on the genetic diseases’ spectrum published in 2011 have been compared to the updated data using the Fisher test, considering 5% as a statistical significance threshold. The data have been stored in a local database for subsequent analyses (Figure 1).

### 2.2. Systematic Review of Literature Focusing on Newborn and/or Carrier Screening for Genetic Diseases among Tunisians

To get knowledge about the current genetic disease screening situation in Tunisia, a selective literature search was carried out. Multiple keywords have been used to develop an advanced PubMed query string as below: 

(“Genetic Diseases, Inborn”[Mesh]) AND ((“Mass Screening”[Mesh] OR “Diagnosis”[Mesh] OR “Maternal Serum Screening Tests”[Mesh] OR “High-Throughput Screening Assays”[Mesh] OR “Neonatal Screening”[Mesh] OR “Multiphasic Screening”[Mesh] OR “Genetic Carrier Screening”[Mesh] OR “Diagnostic Screening Programs”[Mesh] OR “Direct-To-Consumer Screening and Testing”[Mesh] OR “Prenatal Diagnosis”[Mesh] OR “Noninvasive Prenatal Testing”[Mesh] OR “Preimplantation Diagnosis”[Mesh] OR “Mandatory Testing”[Mesh]) AND (Tunis*))

Only studies dealing with genetic disease screening among Tunisians were kept. In addition, we asked referring experts (clinicians, researchers) with whom we collaborate in order to provide more information concerning screening for these disorders. Moreover, grey literature was an important resource in this part of the systematic review. 

Furthermore, we have defined a list of genetic diseases frequently described among Tunisians based on epidemiological data, essentially patients’ counts. The experience feedback of our collaborators was of great value in this step of analysis. We have checked the eligibility of the identified commonly-reported genetic diseases for systematic newborn and/or carrier screening in our country by responding to key questions relative to newborn screening and using the seven ACOG carrier-screening criteria mentioned in their committee opinion in 2017. These criteria are the following (a) well-defined phenotype, (b) detrimental effect on the quality of life, (c) cognitive or physical impairment, (d) surgical or medical intervention is required, (e) an early life onset, (f) availability of prenatal diagnosis, and (g) carrier frequency of 1 in 100 or greater. Evaluation of these criteria was conducted by a genetic counselor.

## 3. Results

In this study, we present the actual situation of genetic diseases in Tunisia. Compared to what has been published in 2011, 243 additional genetic conditions have been identified, with an average of approximately 23 genetic diseases reported every year. Thus, the total number of genetic diseases in Tunisia amounts to 589 clinical and/or genetic entities, in which 41.3% come from the present data mining.

### 3.1. Classification of Genetic Diseases According to WHO-ICD 10

**Figure 2:**
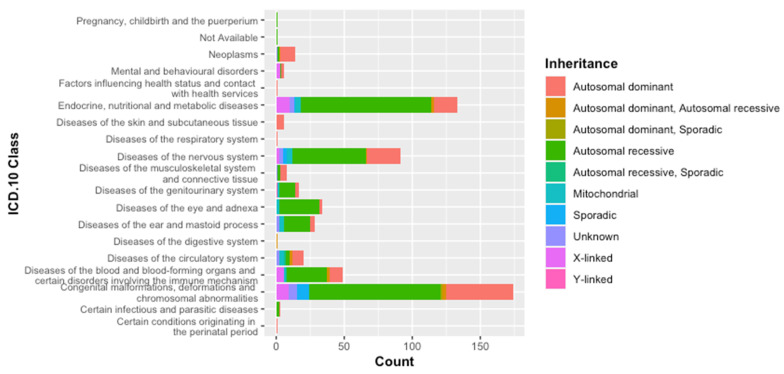
WHO ICD-10 classification of genetic diseases.

We opted for the international medical classification (WHO ICD-10) of genetic diseases described in the Tunisian population. Therefore, such classification revealed that congenital malformations, deformations, and chromosomal abnormalities are the most commonly reported (29.54%). Endocrine, nutritional, and metabolic diseases are the next most frequent group of diseases (22%), followed by diseases of the nervous system (15.45%) (Figure 2). We report a slight increase in the following disease groups: diseases of the ear and mastoid process and diseases of the eye and adnexa. We also noticed that diseases of the circulatory system are highly reported. A comparison between genetic diseases spectrum data of 2011 and the updated data of our study highlighted a significant difference in the distribution of disease classes (Fisher test *p*-value = 9.9 × 10^−4^).

### 3.2. Classification According to the Inheritance Mode 

Among genetic diseases derived from this analysis (243 genetic diseases), autosomal recessive diseases (AR) are the most frequent (54.7%), followed by autosomal dominant (AD) disorders (27.16%). X-linked (XL) conditions were encountered 5% of the time. Diseases transmitted with both AR and AD (AR, AD) are more prevalent than in data reported before 2011. Remarkably, diseases inherited via mitochondrial mode are becoming more reported (8.2%). In order to check for an eventual change in the inheritance mode distribution between the two datasets, we have compared our current data to that of the previous spectrum. We found a significant difference (Fisher’s test; *p*-value = 5 × 10^−4^). Despite this difference, the whole spectrum of genetic diseases described among Tunisians (589 genetic diseases) has kept the same pattern illustrated by the high frequency of AR diseases (60%) followed by the AD conditions (24.3%) (Figure 2).

### 3.3. Molecular Etiology of the Genetic Diseases in the Tunisian Population

**Figure 3:**
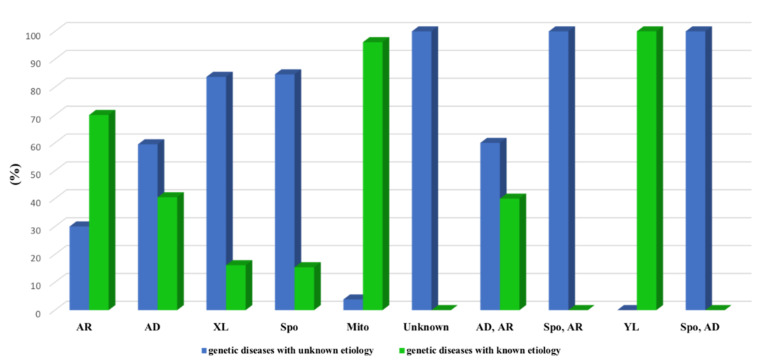
Classification according to the inheritance mode of the whole spectrum of genetic disorders (AR: Autosomal Recessive; AD: Autosomal Dominant; XL: X-linked; Spo: Sporadic; Mito: Mitochondrial; YL: Y-linked).

Sixty-one percent (61 %) of the 589 genetic diseases have a known molecular etiology (358 genetic diseases), meaning that the responsible gene with at least one mutation has been identified among Tunisian patients. About 69% of these diseases are autosomal recessive, 16.2% are autosomal dominant, 7% are caused by a mutation in mitochondrial genes, 6% are X-linked, and only 0.3% are Y-linked or sporadic. Autosomal dominant or autosomal recessive diseases are encountered at 1.2% (Figure 3).

Among these diseases, 21 conditions were of unknown molecular etiology before the last decade (reported in the study of Romdhane and Abdelhak, 2011) (Table 1). Eleven diseases mainly belong to the endocrine, nutritional, and metabolic disorders class. Three are diseases of the nervous system, and one is a bleeding disorder. The eight remaining correspond to congenital malformations, deformations, and chromosomal abnormalities (Table 1). Next-generation sequencing has been used to elucidate the molecular etiology in four of them (Table 1). Referring to the original study conducted by Romdhane and Abdelhak in 2011, the Tunisian mutational spectrum included 420 mutations (98% nuclear, 2% mitochondrial) (Romdhane et al. 2011) (23). Here, the global spectrum comprises more than 896 mutations. These mutations affect 313 genes: 95.5% nuclear (299 genes) and 4.5 % mitochondrial (14 genes). The actual Tunisian mutational spectrum encompasses 429 (48%) founder mutations responsible for 197 genetic diseases. Allelic heterogeneity was noticed for 154 genes (Table S1 Supplementary Data). HBB, G6PD, and CFTR genes were the most mutated genes among Tunisians with at least 25 alterations.

### 3.4. Comorbidity among Tunisians

Comorbidity is defined as the expression of another disease in addition to the primary disease. This phenomenon has been reported among Tunisian families (Romdhane et al., 2016) [42]. In our updated data, we have identified fourteen co-occurrences of multiple diseases since 2016 (Table 2). All of them belong to the genetic disease–genetic disease class of comorbid associations (in other words, both diseases are caused by a mutation in a single gene or multiple genes) with a consanguinity history among six of them. Both familial (6) and individual (8) comorbidities have been identified. They are either with the same mode of transmission or not. The comorbid associations include diseases of the same pathological group, for example, genodermatoses (Ichthyosis congenital and Erythrokeratodermia variabilis, Xeroderma pigmentosum group C, and Rothmund Thomson syndrome) or not (Table 2).

### 3.5. Phenotypic Features of Genetic Diseases in Tunisia

Atypical phenotypes are also reported and characterize several genetic diseases among Tunisians. By atypical clinical presentations, we mean unusual symptoms of the disease. Chanarin–Dorfman syndrome, palmoplantar keratoderma, juvenile Parkinson’s disease, and multiple endocrine neoplasia type 2A were described in Tunisian patients with unusual presentations (Table 3). 

### 3.6. Epidemiology of Genetic Diseases in Tunisia

For epidemiological parameters, we focused on the frequency of the whole reported disease spectrum by targeting either the prevalence or the incidence. We noticed the paucity of such epidemiological data as only 26 (4.6%) genetic diseases described among Tunisians have defined prevalence and/or incidence (Table 4). For some genetic disorders (five diseases), the epidemiological data are described among specific ethnic groups or in specific regions. For the remaining diseases, only the number of studied patients and/or families is reported. In an attempt to compare these epidemiological data, we tried to use the same scale of a rare disease threshold prevalence in Europe (1/2000). By aligning to this definition, we found that seven diseases largely exceed this threshold. For ten others, the available epidemiological data suggests that they are ultra-rare diseases (Table 4).

### 3.7. Current Situation of Newborn and/or Carrier Screening for Genetic Diseases in Tunisia

On 1 July 2021, the second PubMed-MeSH query described above yielded 732 results, of which 26 articles have been retained for further analysis. Three articles (in French) found in Google Scholar have also been included. Ultimately 29 articles were screened. In all of these studies, the authors highlight the key role of screening for genetic diseases as a state-run health program in Tunisia. However, neonatal newborn and carrier screenings were limited to pilot experiments for only six conditions, and they have not been replicated or extended geographically to the level of the country. Systematic newborn screening for hearing impairment is exclusively conducted in the governorate of Sfax in the south (Table 5). In fact, 16 disorders are likely frequent among Tunisians, of which 10 are part of the Recommended Uniform Screening Panel. All of them are associated with founder and/or recurrent mutations, and 14 met all the 7 ACOG criteria, with 2 late-onset diseases (Table S2 Supplementary data).

## 4. Discussion

In this study, we attempted to provide a comprehensive and up-to-date overview of the genetic disease spectrum described among Tunisians. Our findings showed that the number of reported genetic conditions increased from 346 to 589 in the last eleven years (2010–2021). This highlights remarkable progress in the availability of molecular diagnosis of genetic diseases in Tunisia. The development of biomedical health research activities, the building capacities in this field, and the use of high-throughput sequencing technologies facilitated by international collaborations, have played a critical role in this progress [7].

Genetic disorders present a health burden, especially among inbred populations, including Arab countries and several African communities [9,75,76]. Here, in the recent spectrum, most of the genetic diseases described among Tunisians are autosomal recessive (59.8%). A slight reduction is noticed compared to data reported in 2011; however, their frequency is still high, which could be explained by the socio-cultural features in the Tunisian population [19]. Indeed, consanguineous marriages, with an extreme predominance of first-cousin marriages associated with large family sizes, are responsible for the high prevalence of recessively inherited conditions among Tunisians [20,22,23,77]. It has been proven that there is an increased risk of almost eight times for expressing a recessive disease among first-cousin marriage’s progeny [22].

Another impact of the elevated rate of consanguinity and inbreeding is the co-occurrence of two or more phenotypes within the same family [42]. Comorbidity, described particularly in inbred populations, makes genetic counseling and prenatal diagnosis challenging [42,44]. The study of Romdhane and her colleagues revealed 75 genetic disease co-occurrences identified in Tunisian patients. Thirty-nine (39) of them have been reported among Tunisian consanguineous families [42]. Since then, 14 additional comorbidities of genetic diseases in Tunisians were reported in our study, for which NGS techniques had largely contributed to the molecular diagnosis. Consanguinity was noted in six of them. 

Several variants, either in the same gene or in related genes, were correlated with the high frequency of genetic diseases in inbred communities [76]. In the current report, we have found that the Tunisian mutational spectrum includes more than 896 mutations affecting 313 genes. Among these mutated genes, allelic heterogeneity was identified for 154 of them. Such characteristics render the diagnosis difficult and could lead to misdiagnosis, especially in developing countries, such as Tunisia [7,10]. Screening for founder mutations among endogamous populations is a cost-effective molecular diagnosis strategy [10], but it may be inefficient when applied to atypical rare phenotypes and comorbidities (Mezzi et al., Unpublished). Consequently, whole-exome sequencing (WES), could be an alternative choice to elucidate the molecular etiology of such conditions and is always recommended, especially when targeted mutation screening and/or targeted gene sequencing results are negative or ambiguous [78,79]. In Tunisia, molecular investigation of genetic diseases is mainly conducted using Sanger DNA sequencing. However, since 2013, we note that next-generation sequencing (NGS) has become more affordable to the scientific community, mainly through international collaborations. We found that the use of high-throughput sequencing has led to the identification of the molecular bases of at least 45 mendelian diseases described in Tunisians.

Autosomal dominant diseases were found in 24.5% of reported Tunisian patients. Although intrafamilial marriages have no important effect on the prevalence of autosomal dominant diseases, significant consequences on the expression of such disorders could not be completely excluded [7]. Indeed, consanguineous unions could increase the likelihood of homozygosity for dominant genetic variations [20,80], which could induce more severe phenotypes and even atypical ones [20,80]. This was the case for a large consanguineous Tunisian family with granular corneal dystrophy type I in which homozygous members for a *TGFBI* mutation were the severely most affected [81]. Allelic comorbidity involving a dominant disease illustrated by the consanguineous family reported by Sfar et al., is also an excellent example of such an effect [82]. The parents, who presented a mild phenotype of neonatal hyperparathyroidism, were heterozygous for a *CASR* gene mutation. Their daughter had a severe hyperparathyroidism phenotype because of her homozygous state for the same mutation [82].

Accurate prevalence and incidence rates are crucial to developing an understanding of a disease’s natural history and population burden [83]. However, among the 589 genetic disorders described among Tunisians, only 26 (4.6%) have a defined prevalence and/or incidence yet without systematic updates. Some genetic diseases, including familial hypercholesterolemia (12.12/2000) and hemoglobinopathies (89.6/2000), are particularly frequent in Tunisia compared to Europe, where they are reported as rare diseases. Several others are rare and even ultra-rare, depending on the defined threshold. The scarcity of accurate epidemiological data could lead to misdiagnosis [84] and indicates the necessity of comprehensive epidemiology and patient registry creation [7,20,85]. Thus far, only three patient registries have been set for genetic disorders in Tunisia, namely, Gaucher Disease (http://www.maladie-gaucher-tunisie.org/, accessed on 24 September 2021), Fanconi Anemia (http://fanconi-tunisie.net/, accessed on 24 September 2021), and bleeding disorders [86]. Furthermore, they are not systematically updated, and they are facing personal patient data security problems and storage systems issues. Building rare genetic diseases registries in developing countries is challenging [84,86]. The main obstacle is the drastic lack of well-defined policies and the paucity of common protocols for these registries [84]. Moreover, data on rare genetic diseases are usually unharmonized (archived in hand-written form or in simple excel files). Consequently, data treatment and handling are time-consuming tasks [85]. In addition, the funding needed for the infrastructure of these registries could be challenging, as not all countries can cover their costs [85]. Considering the fact that epidemiological studies of rare and extremely rare diseases are difficult to set at the level of the country (as for classical epidemiological studies), we highly recommend national registries to establish either specific to each disease or to a group of diseases in order to have not only a better estimation of their figures but also for better care interventions. Moreover, developing a population-specific genetic diseases database is undoubtedly needed to have a complete overview of their spectrum in a given population. This informative online database will be a hopeful starting point for healthcare experts and researchers [87].

Molecular data availability on relatively frequent genetic rare diseases in inbred populations could be an ideal way to boost current research focusing on targeted therapies, including gene and mutation-specific therapies, hence leading to the implementation of precision medicine. Several diseases identified in the Tunisian population are subject to targeted therapy based on their genetic information. Bloom syndrome (BS) is a DNA-repair disease characterized by a high risk of cancer at an early age [88]. It has been established that a correct diagnosis should be clearly set before using chemotherapy in BS patients because drug dose is highly dependent on the disease severity degree [88] while conventional chemo or radiotherapy would lead to a profound genomic instability and, consequently, enhanced carcinogenesis. In the same way, knowing the gene or the mutation is also fundamental for implementing efficient therapy. Indeed, improved cochlear implant outcomes have been observed among deaf Tunisian individuals for whom the hearing loss is related to GJB2 mutations and who were implanted at an early age [89,90]. Drugs targeting mutations are an innovative therapeutic approach in cystic fibrosis (CF) [91,92]. CF is a common genetic life-shortening condition in the Caucasian population, and it is not so rare in North Africa [93]. The *CFTR* gene has accumulated multiple founder mutations that could be used as a target for personalized therapy [94]. Lumacaftor was tested as an effective mutation-specific therapy for African-American CF patients targeting the shared founder mutation that is also reported in the Tunisian population [95]. Gene therapy is a promising strategy for the permanent treatment of genetic diseases. LGMD2C has the highest incidence in North Africa, and it is caused by the predominant founder deletion c.521delT in the SGCE gene [96,97]. A gene therapy trial has been performed targeting patients with these founder mutations as a crucial inclusion criterion [98]. Nine Tunisian patients were involved in this trial [98]. With mutation-specific therapies being under development, a correct diagnosis is more than ever mandatory for assessing whether patients are eligible or not for adequate treatments.

The increased focus on genetic diseases in Tunisia over the last decades has been spurred mainly by the clinical and molecular investigations intended to improve the diagnosis. Nevertheless, genetic services are lacking and are not equally distributed across the country. Indeed, only three main medical genetic services are available: Charles Nicolle hospital in Tunis, Farhat Hached hospital in Sousse, and Habib bourguiba hospital in Sfax. A unit for congenital and inherited diseases was recently built at the “Mongi Slim hospital” in La Marsa, providing cytogenetic tests. Furthermore, breast and cervical cancer screening has been integrated into basic health care services within the framework of national cancer control plans (Ministère de la Santé, Direction de Santé, Plan pour la lutte contre le cancer 2015–2019) (Tunis: Ministère de la Santé, 2015). In addition, the establishment of an NGS platform in Institut Pasteur de Tunis, allowed the setting up of molecular diagnosis of familial cancers, thus contributing to the better characterization and widening of the mutation spectrum of BRCA1/2 genes in the Tunisian population [99]. The increased prevalence of cystic fibrosis (CF) among Tunisian children and the potentially severe complications associated with this disease justify the management of CF by a multidisciplinary team of clinicians in the child pulmonology referral unit in Bechir Hamza children’s Hospital of Tunis [54]. In this unit, englobing the largest national series, patients are managed by several therapeutic strategies (respiratory physiotherapy, antibiotherapy, aerosol therapy, vitamin therapy, inhaled corticosteroids, etc.) [54,94]. Genetic counseling and prenatal diagnosis are also proposed to families [54]. This example illustrates the importance of the referral centers for better knowledge of the disease where adequate clinical and molecular competencies are available, but it is still insufficient as the commitment of the national authorities is required in order to support these centers by providing adequate technological platforms.

From a public health perspective, experts and policymakers have opted that newborn and carrier screening should be conducted as a state-run program [100,101]. This will allow early detection of genetic conditions and congenital disorders, leading to reduced health disparities and mortality at a young age [102,103]. The infant mortality rate is an indicator of a state’s health, and reducing this rate is an explicit goal of sustainable development (2030 United Nations Sustainable Development Goals). Several genetic conditions are recommended to be screened among newborns and/or at-risk individuals around the world. Among these, we have the Recommended Uniform Screening Panel (RUSP) issued by the U.S. Health and Human Services (HSS) Federal Advisory Committee on Heritable Disorders in Newborns and Children (ACHDNC). The RUSP includes 35 core conditions (for which newborn screening is highly recommended) and 26 secondary conditions (for which newborn screening is optional). Furthermore, a data-driven evaluation has been conducted in which an Expanded Carrier Screening (ECS) panel of 172 conditions have been defined and met all the required criteria defined by the American College of Obstetricians and Gynecologists [104,105]. In the MENA region, reducing genetic condition expression, particularly those recessively inherited, is a salient issue mainly due to their high prevalence because of inbreeding and the severe expression in newborns [106,107]. However, among several countries of this region, newborn screening is not introduced systematically as a health policy, and it is only limited to pilot projects [106,108]. In fact, only Gulf states have a newborn screening panel for 20 metabolic conditions [106,109]. Egypt and Palestine also have a national neonatal screening plan for congenital hypothyroidism and phenylketonuria [110,111,112]. Of the disorders recommended to be screened (RUSP and ECS panel), 49% are described among Tunisian patients (Table S3 Supplementary Data), representing 19% of the genetic disease spectrum in Tunisia and could be considered, therefore, as a potential target for intervention. The absence of such an intervention in Tunisia could be explained by the inability of the Ministry of Health and of the social security system to ensure adequate care or health coverage for individuals screened as positive [108,113]. Consequently, we propose, instead of systematic neonatal screening, to conduct cascade carrier screening with specific regional programs that take into account the most frequent diseases and underlying deleterious founder alleles. This helps to decrease morbidity and mortality, as well as to improve the quality of life and life expectancy for patients with genetic diseases.

A comprehensive genetic education and premarital genetic counseling programs can also help to reduce the burden of genetic diseases in inbred communities [114,115]. In Bahrain, educational programs aimed at high school children have had a marked beneficial effect in lessening the incidence of sickle cell disease [116]. It has been suggested that raising public awareness of sickle cell disease through prevention campaigns using informational programs with different communication media would be efficient to reduce the burden of the disease in the population in addition to programs that have proven their efficiencies, such as student screening programs, premarital counseling, and newborn screening [117].

Patient support groups could act as key support for rare genetic diseases. As stated earlier, hearing loss constitutes a frequent disease group in our population. In an attempt to improve the auditory health in the Tunisian population, hearing screening and awareness-raising campaigns are being organized by NGOs, such as ICHARA, in the frame of a science shop project participatory research support [118].

## 5. Conclusions

Our study provides a better knowledge of the genetic diseases spectrum in Tunisia, which is useful for this country, as well as for neighboring countries, in order to improve the diagnosis, to reduce misdiagnosis, and therefore, patients will have earlier and better management. This would also help to avoid possible complications and reduce the costs of managing these diseases. Molecular and genetic data for these diseases are becoming more widely available through the development of biomedical health research activities and access to high-throughput next-generation sequencing. However, the Tunisian population still faces challenges, especially in the case of dual diagnosis and co-occurrence in the same patient or family, which delays the appropriate patient management. Taking this into account, in addition to the relatively high genetic and mutational heterogeneity, we recommend the creation of diagnostic referral centers that leverage some technologies, such as whole-exome sequencing, as a first intention tool for genetic disease elucidation in the country. These specialized structures could potentially offer accurate molecular diagnostic services at a lower cost and allow faster, efficient patient management. Furthermore, patient management should include multidisciplinary team investigators working in a well-defined care pathway. Vigilant monitoring of emerging clinical manifestations is recommended, especially in the case of genetic disorders. Furthermore, studies are needed to accurately set the epidemiological data of genetic diseases aiming to implement better and appropriate healthcare measures. Patient support groups have a significant role to play in promoting patient voice and influencing the decision-makers.

## Figures and Tables

**Table 1:** Diseases with an identified molecular etiology determined in the last decade.

Disease Name	OMIM	Inheritance	Gene (OMIM)	Genetic Variant(s)	ICD-10 Classification	Approach Used for Molecular Diagnosis	References
Adrenal Hyperplasia, Congenital, due to 11-β-Hydroxylase Deficiency	202010	AR	*CYP11B1* (610613)	c.1066C > T (p.Gln356Ter)	Endocrine, nutritional, and metabolic diseases	Sanger sequencing	[24]
				c.1136G > T (p.Gly379Val)			
Adrenoleukodystrophy	300100	XL	ABCD1 (300371)	c.284C > A (p.Ala95Asp)	Endocrine, nutritional, and metabolic diseases	Sanger sequencing	[25]
Alstrom syndrome	203800	AR	*ALMS 1* (606844)	c.10388-2A > G	Congenital malformations, deformations, and chromosomal abnormalities	Targeted Gene sequencing	[26]
Bloom syndrome	210900	AR	*BLM* (604610)	c.1980-1982delAA (p.Lys662fsX5)	Congenital malformations, deformations, and chromosomal abnormalities	Sanger sequencing	Ezzine et al., Unpublished [27]
FVII deficiency	227500	AR	*F7* (613878)	c.90-91insA (p.12LeufsX11)	Diseases of the blood and blood-forming organs and certain disorders involving the immune mechanism	Sanger sequencing	[28]
				c.592 T > C (p.Cys198Arg)			
				c.1615 G > C (p.Gly539Arg)			
				c.2409 T > C (p.Asn784Asn)			
				c.2167 G > A (p.Ala704Thr)			
				c.3870-3871insA (p.1271LysfsX29)			
				c.1696 C > T (p.Leu547Phe)			
				c.1492 G > A (p.Gly479Arg)			
				c.77 T > C (p.Leu7Pro)			
				c.5071-5075delATGAA (p.1671-3fsX)			
				c.3637-3638delA (p.1191IlefsX5)			
				c.6873-6876delTC (p.2272ThrfsX)			
				c.4379-4380insA (p.1441LysfsX2)			
				c.3637-3638insA (p.1191LeufsX29)			
				c.2236-2237insT (p.727SerfsTer)			
Gaucher disease type III A	231000	AR	*GBA* (606463)	c.1109T > C (p.Phe370Ser)	Endocrine, nutritional, and metabolic diseases	Sanger sequencing	[29]
				c.1330_1331delGAinsCC (p.Asp444Pro)			
Junctional epidermolysis bullosa (JEB) Herlitz type	226700	AR	LAMA3 (600805)	c.2865C > G (p.His955Gln)	Congenital malformations, deformations, and chromosomal abnormalities	Sanger sequencing	[30]
Lafora disease	254780	AR	*EPM2A* (607566)	c.659 T > A (p.Leu220Gln)	Diseases of the nervous system	Sanger sequencing	[31]
Lysosomal acid lipase deficiency	278000	AR	*LIPA* (613497)	c.153 C > A (p.Tyr51Ter)	Endocrine, nutritional, and metabolic diseases	Sanger sequencing	[32]
Maple syrup urine disease, type Ib	248600	AR	*BCKDHB* (248611)	c.716A > G (p.Glu239Gly)	Endocrine, nutritional, and metabolic diseases	Sanger sequencing	[33]
Meckel syndrome type 2	603194	AR	*TMEM216*(613277)	c.341T > G (p.Leu114Arg)	Congenital malformations, deformations, and chromosomal abnormalities	Exome sequencing	[34]
MELAS syndrome	540000	Mitochondrial	*MT-TV* (590105)	m.1640A > G	Diseases of the nervous system	Sanger sequencing	[35]
Microphthalmia isolated, 6	613517	AR	*PRSS56* (613858)	c.1059_1066insC (p.Gln356ProfsTer152)	Congenital malformations, deformations, and chromosomal abnormalities	Sanger sequencing	[36]
Miyoshi muscular dystrophy 1	254130	AR	*DYSF (603009)*	c.4597-2A > G	Diseases of the nervous system	Targeted Gene Sequencing	[37]
Mucopolysaccharidosis type II	309900	XL	*IDS* (300823)	c.240 + 1 G > A	Endocrine, nutritional, and metabolic diseases	Sanger sequencing	[38]
				c.263G > A (p.Arg88Pro)			
				c.610C > T (p.Gln204Ter)			
				c.1348G > A (Asp450Asn)			
				c.281G > A (p.Gly94Asp)			
				c.1186C > T (p.Gln396Ter)			
Mucopolysaccharidosis type IIIA	252900	AR	*SGSH* (605270)	c.2t > C (p.Met1Thr)	Endocrine, nutritional, and metabolic diseases	Sanger sequencing	[39]
				c.1129C > T (p.Arg377Cys)			
				g.75802301_75809393del			
				c.1093C > T (p.Gln365Ter)			
				c.29dup (p.Leu11AlafsTer22)			
				c.197C > G (p.Ser66Trp)			
				c.1080del (p.Val361SerfsTer52)			
Mucopolysaccharidosis type IIIB	252920	AR	*NAGLU* (60970)	c.1674C > G (p.Tyr558X)	Endocrine, nutritional, and metabolic diseases	Sanger sequencing	[39]
				c.1811C > T (p.Pro604Leu)			
Mucopolysaccharidosis type IIIC	252930	AR	*HGSNAT* (610453)	c.1209G > A (p.Trp403X)	Endocrine, nutritional, and metabolic diseases	Sanger sequencing	[39]
				c.1880A > G (p.Tyr627Cys)			
Myoclonic epilepsy of Lafora	254780	AR	*EPM2A* (607566)	c.659 T > A (p.Leu220Gln)	Diseases of the nervous System	Sanger sequencing	[31]
Nephropathic cystinosis	219800	AR	*CTNS* (606272)	c.1515G > A (p.Gly308Arg)	Endocrine, nutritional, and metabolic diseases	Sanger sequencing	[40]
				c.771_793del (p.Gly258SerfsTer30)			
Punctate palmoplantar keratoderma type 1	148600	AD	*AAGAB* (614888)	c.481C > T (p.Arg161Ter)	Congenital malformations, deformations, and chromosomal abnormalities	Whole exome sequencing	[41]

AR: Autosomal Recessive; AD: Autosomal Dominant; XL: X-linked; Mito: Mitochondrial.

**Table 2:** Comorbid associations described among Tunisians between 2016 and 2020.

Associations	Familial/Individual	Inheritance	Consanguinity	References
Allgrove syndrome—Hearing loss	Familial	AR—AR	Yes	Mkaouar et al., unpublished
Amyotrophic lateral sclerosis—Behcet’s disease	Individual	AD, AR—Unknown	NA	[43]
Autism—Hearing loss	Familial	Complex heredity—AR	Yes	Lahbib et al., unpublished
Cutis laxa—Pulmonary disease	Familial	Unknown-Unknown	NA	Tinsa et al., unpublished
Growth hormone deficiency—Immunodeficiency	Individual	Unknown-Unknown	NA	Tinsa et al., unpublished
Ichthyosis congenital autosomal recessive 1—Erythrokeratodermia variabilis	Familial	AR—AD	No	Laroussi et al., Unpublished
Ichthyosis congenital autosomal recessive 5—hearing loss	Individual	AR—AR	Yes	[44]
Ichthyosis congenital autosomal recessive 1-Muscular dystrophy limb girdle type 2A	Individual	AR-AR	Yes	Mezzi et al., Unpublished
*Incontinentia pigmenti*—Noonan syndrome	Individual	XLD—AD	No	[45]
Maternally inherited diabetes—deafness-Retinopathy	Individual	Mitochondrial—Unknown	No	[46]
Niemann-Pick disease type B—Systemic lupus erythematous	Familial	AR—AD	No	[47]
Pernicious anemia—Pseudohypoparathyroidism	Individual	Unknown-Unknown	NA	Tinsa et al., unpublished
*Xeroderma pigmentosum* group A—Autoimmune polyendocrinopathy syndrome I	Individual	AR—AR, AD	Yes	Messaoud et al., Unpublished
*Xeroderma pigmentosum* group C—Rothmund Thomson syndrome	Familial	AR—AR	Yes	Ezzine et al., Unpublished

AR: Autosomal Recessive; AD: Autosomal Dominant; XLD: X-linked Dominant; Mito: Mitochondrial.

**Table 3:** Genetic diseases with unusual clinical findings among Tunisians.

Diseases	Unusual Clinical Findings	Case count	References
Chanarin-Dorfman syndrome	Thyroid function impairment	7	[48]
Palmoplantar keratoderma	Abnormal cornification and a diffuse yellowish keratoderma with the characteristic skin thickening	1	[49]
Juvenile Parkinson disease	No evidence of sleep or autonomic dysfunctions and psychiatric disorders in both patients	1	[50]

**Table 4:** Genetic diseases with available epidemiological data among Tunisians.

Genetic Disease (MIM)	Frequency	References	Measure of Estimation	State/Region/Group	Prevalence in Orphanet **
Anemia, Nonspherocytic hemolytic, due to G6PD deficiency (ANH- G6PD) (300908)	18,400/million/year	[51]	Incidence	All across Tunisia	<1/1,000,000
β thalassemia ( β -thal) (613985)	44.2/2000	[52]	Prevalence	All across Tunisia	1–9/1,000,000
Creutzleldt-Jakob Disease (CJD*) (123400)	2.3/million/year	[53]	Incidence	Among Tunisian Jews	<1/1,000,000
Cystic fibrosis (CF*) (219700)	1.5 new cases/year 0.4/1000	[54]	Incidence Prevalence	In the Pediatric department B of the Children’s Hospital Béchir Hamza de Tunis (among patients’ series) Most from the north and the south of Tunisia	NA
	0.4/1000 Its prevalence was 0.4 per 1000 hospitalizations.				1–9/100,000
Dermatitis, Atopic (ATOD) (603165)	451 cases during a 7 years period	[55]	Incidence	All across Tunisia	NA
Epidermolysis bullosa dystrophica Hallopeau-Simens type 1 (RDEB*) (226600)	2.3/2000	Cherif et al., 2005 unpublished	Prevalence	All across Tunisia	<1/1,000,000
	0.1/2000	[56]		In the governorate of Sfax	
Exfoliation syndrome (XFS) (177650)	220/2000	[57]	Prevalence	All across Tunisia	NA
Familial Adenomatous polyposis of the colon (FAP1) (MIM: 175100)	74/million/year	[58]	Incidence	All across Tunisia	1–9/100,000
Fanconi anemia (FA) (227650)	1.4/million/year	[59]	Incidence	All across Tunisia	1/160,000
Familial hypercholesterolemia, (FHCL*)	12.12/2000	[60]	Prevalence	In central and southern Tunisia	1–9/1,000,000
Gaucher disease, type I (GD1) (230800)	0.0096/2000	[61]	Prevalence	All across Tunisia	1/100,000
Glycine encephalopathy (GCE*) (605899)	1/9322	[62]	Incidence	In the governorate of Kairouan	1–9/1,000,000
Glycogen storage disease type Ia (GSD1A*) (232200)	0.02/2000	[63]	Prevalence	In the north of Tunisia	NA
	7.93/million/year		Incidence		1/100,000 (Incidence)
Hemoglobinopathies	89.6/2000	[52]	Prevalence	All across Tunisia	NA
Hurler syndrome (HS*) (607014)	0.064/2000	[64]	Prevalence	In Tunisian Jews	1/200,000
Limb-girdle Muscular dystrophytype 2C (LGMD2C) (253700)	0.6/2000	[65]	Prevalence	All across Tunisia	1–9/1,000,000
Lynch syndrome 1 (LS1) (120435)	70/million/year	[66]	Incidence	All across Tunisia	NA
Megaloblastic anemia 1 (MGA1*) (261100)	2/2000	[67]	Prevalence	In Tunisian Jews	NA
Mucopolysaccharidosis I (MPS1-S) (607016)	0.0126/2000	[59]	Prevalence	All across Tunisia	1/100,000
Mucopolysaccharidosis type IIIA (MPS 3A) (252900)	0.007/2000	[59]	Prevalence	All across Tunisia	1–9/100,000
Mucopolysaccharidosis type IVA (MPS4A) (253000)	0.025/2000	[68]	Prevalence	All across Tunisia	1–5/10,000
Mucopolysaccharidosis type VI (MPS 6) (253200)	0.013/2000	[62]	Prevalence	All across Tunisia	1–9/1,000,000
Niemann Pick disease B (607616)	0.1/2000	[66]	Prevalence		1–9/1,000,000
Phenylketonuria (PKU) (261600)	Varies between 0.29/2000 and 0.6/2000	[67]	Prevalence	All across Tunisia	1–5/10,000
Sickle Cell Anemia (SCA) (603903)	37.8/2000	[52]	Prevalence	All across Tunisia	1/150
*Xeroderma pigmentosum* , complementation group A (XPA) (278700)	0.2/2000	[69]	Prevalence	All across Tunisia	1/1,000,000

AD: Autosomal dominant, AR: Autosomal recessive, XLD: X-linked dominant. * Diseases with prevalence and/or incidence among a specific ethnic group or in specific regions. ** (https://www.orpha.net/, accessed on 27 September 2021).

**Table 5:** Screening of genetic diseases in Tunisia.

Diseases (MIM)	Screening Type	Screening Techniques	Country Coverage	References
α-thalassemia	Prenatal screening	DNA analysis from amniotic fluid	Biochemistry and molecular biology department in children’s hospital of Tunis (pilot study)	[70]
β-thalassemia	Prenatal screening Carrier screening	DNA analysis from amniotic fluid Screening for mutations described in Tunisians	Biochemistry and molecular biology department in children’s hospital of Tunis (pilot study)	[70]
Congenital hypothyroidism	Newborn screening	TSH and T4 radioimmunoassay on drops of blood	Maternity and Neonatal Centre in Tunis (pilot study)	Elkadri et al. *
Cystic fibrosis	Prenatal screening	Genetic analysis by denaturant gradient gel electrophoresis and denaturing high-pressure liquid phase chromatography	Biochemistry Laboratory, Bechir Hamza Children’s hospital in Tunis Center of maternity and neonatology, in Tunis (pilot study)	[71]
Hearing impairment	Newborn screening	Evoked otoacoustic emissions (EOAE) and auditory brain stem response (ABR)	Charles Nicolle hospital of Tunis ((pilot study) Regional hospital in Nabeul (pilot study) Regional hospital in Sfax (systematic)	[72] Feedback from collaborating clinician
G6PD deficiency	Newborn screening	Dosage of the enzymatic activity using spectrophotometric method	Maternity and Neonatal Centre in Tunis (pilot study)	[73]
Phenylketonuria	Newborn screening	Dosage of phenylalanine in dried blood spots	Hospital of La Rabta in Tunis (pilot study)	[68]
Sickle cell disease	Newborn screening	Isoelectrofocusing	Maternity Centre of Aziza Othmana Hospital and Neonatal and Maternity Centre—La Rabta in Tunis (pilot study)	[74]

* Available online: http://docplayer.fr/66862824-Depistage-de-l-hypothyroidie-congenitale-a-tunis-seuils-de-rappel-et-protocole.html (accessed on 27 September 2021).

## Data Availability

Not applicable.

## Supplementary Materials

The following are available online at https://www.mdpi.com/article/10.3390/genes12111820/s1, Table S1: Mutated genes among Tunisians, Table S2: Frequent genetic diseases in Tunisia, Table S3: Genetic diseases screened recommended for newborn and/or carrier screening.
