# Supplementary material for: Spectrum of Genetic Diseases in Tunisia: Current Situation and Main Milestones Achieved
Source: Genes (Basel). 2021 Nov 19;12(11):1820. doi: 10.3390/genes12111820 (PMC8617973; doi:10.3390/genes12111820)
Supplement: Supplementary file 1 [file genes-12-01820-s001.zip › Table S1 supplementary data.pdf]

Table S1 Supplementary data: Mutated genes among Tunisians

Gene Symbol	**Number of Mutations among Tunisians**
AAAS	1
AAGAB	3
ABCA4	2
ABCD1	4
ABHD5	1
ADAMTS13	1
ADAMTS17	1
AGA	1
AGL	12
AGPAT2	2
AGXT	14
AHI1	1
AICDA	3
ALB	1
ALDH5A1	1
ALDH7A1	2
ALMS 1	1
ALPL	1
ALS2	1
ALSIN	1
AMN	1
APC	6
APOA5	1
APOB	2
APTX	2
AQP2	1
AR	3
ARSA	2
ARX	2
ASAH1	1
ATM	1
ATP2A2	9
ATP6V0A4	4
ATP6V1B1	6
ATP7B	3
ATXN2	11

AURKC	1
B3GLCT	1
B4GALNT1	2
BBS1	5
BBS10	1
BBS2	2
BBS4	2
BBS5	3
BBS7	1
BCKDHB	1
BLM	4
BMP15	1
BRAF	2
BRCA1	6
BRCA2	5
BSCL2	2
BTK	2
CA2	1
CAPN1	1
CAPN3	3
CASR	2
CD40	1
CD40LG	4
CDKN2A	1
CEP290	3
CERKL	1
CERS3	2
CFI	3
CFP	1
CFTR	25
CHRNE	1
CIB2	1
CLCN1	1
CLDN16	3
CNGA3	1
CNGB1	1
CNGB3	1
COL7A1	18
CRB1	2

CSTB	1
CTNS	2
CTSK	1
CUL7	3
CYBA	1
CYP11B1	4
CYP1B1	5
CYP21A2	18
CYP24A1	2
CYP7B1	4
DBT	1
DDB2	1
DFNB31	1
DGUOK	1
DNAH1	1
DOK7	1
DYM	1
EDNRB	1
EIF2AK3	1
EPB42	1
EPM2A	1
EPS8	1
ERCC2	1
ERCC8	2
ESRRB	1
EXT1	1
EYS	2
F13A1	2
F2	1
F5	2
F7	5
F8	12
FAM161A	1
FANCA	5
FAS	1
FERMT1	1
FGA	2
FGB	1
FGD4	1

FGF23	1
FKRP	4
FLNC	3
FMR1	1
FOXL2	5
FOXP3	1
FXN	1
G6PC	2
G6PD	25
GABRD	1
GALNS	4
GAMT	1
GAN	5
GBA	7
GBA2	4
GDAP1	9
GJB2	21
GJB6	2
GJC2	1
GNAT2	2
GNE	1
GP1BB	3
GPR98	1
GUCY2D	10
HBA1	4
HBB	31
HEXA	1
HFE	2
HGSNAT	2
HOGA1	3
HPRT1	1
HRPT2	1
HSD17B3	1
HSF4	1
HSPG2	4
IDUA	11
IFNGR1	1
IL12B	1
IL12RB1	1

IL12RB2	1
INSR	2
IT15	1
ITGB2	1
KCNJ11	1
KIT	1
KRT5	1
KRT85	1
L2HGDH	2
LAMA2	4
LAMA3	1
LAMB2	3
LDLR	13
LHFPL5	1
LIPA	1
LMAN1	2
LRP5	1
LRRK2	1
LRTOMT	3
MAPRE2	1
MCFD2	3
MECP2	19
MEFV	8
MKKS	3
MLC1	2
MLH1	5
MPZ	1
MSH2	5
MT-CO1	3
MT-CO2	1
MT-CO3	1
MT-CYB	2
MT-ND1	4
MT-ND5	1
MT-RNR1	3
MT-TE	1
MT-TI	2
MT-TL1	1
MT-TW	2

MTATP6	5
MTATP8	2
MTCO3	1
MTTP	7
MUTYH	3
MYBPC3	3
MYH7	7
MYL3	1
MYO15A	4
MYO7A	4
NAGLU	2
NAIP	3
NCF1	1
NCF2	2
ND1	1
ND5	1
NHS	1
NLRP7	4
NOTCH3	1
NPHP1	1
NPHP4	1
NPHS1	4
NPHS2	2
NR0B1	2
NR2E3	1
PAH	8
PAK3	1
PARK2	1
PAX3	4
PCSK9	1
PDE6A	1
PDE6B	3
PEX26	1
PGM3	5
PHGDH	1
PINK1	4
PKLR	6
PMP22	1
POLH	5

POLR3A	1
PQBP1	1
PRNP	1
PROKR2	2
PROM1	1
PROP1	2
PRSS56	1
PSEN1	1
PTPN11	5
PYGM	1
QDPR	1
RB1	3
RECQL4	1
REN	1
RET	1
RFXANK	2
RGS6	1
ROBO3	4
RPE65	5
SACS	8
SAR1B	1
SBF2	1
SCN1A	1
SCN1B	1
SERPINC1	1
SETX	5
SF1	1
SGCA	1
SGCB	1
SGCG	3
SGSH	7
SH3TC2	1
SIX1	1
SLC12A3	1
SLC14A1	1
SLC19A2	1
SLC26A4	3
SLC29A3	1
SLC39A4	6

SLC7A7	1
SLURP1	3
SMN1	9
SMPD1	1
SOS1	2
SPAST	2
SPG11	4
SPTA1	4
SULT2B1	1
TACSTD2	2
TAT	4
TBX22	1
TBXAS1	2
TGM1	2
TGM5	1
TMC1	4
TMEM126A	2
TMEM216	1
TMPRSS3	1
TMPRSS4	1
TNNC1	1
TP53	1
TPO	1
TRIM37	1
TRVAAC1-4	1
TTC8	2
TTPA	1
TYR	3
UBE3A	2
UGT1A1	1
UROD	1
USH1G	1
USH2A	6
USP9Y	4
VDR	4
VKORC1	3
WNT4	1
WT1	1
XPA	5

XPC	3
ZFYVE26	3
ZNF408	1
ZNF469	1
